# Measurement of the Environmental Impact of Materials

**DOI:** 10.3390/ma15062208

**Published:** 2022-03-17

**Authors:** Franz-Georg Simon, Ute Kalbe

**Affiliations:** BAM Bundesanstalt für Materialforschung und-prüfung, 12200 Berlin, Germany; ute.kalbe@bam.de

Global material use has increased by a factor of eight in the 20th century, and has reached more than 10 tons per capita per year [1]. Negative impacts on the environment can occur in all phases of a material’s life cycle, i.e., from its production from natural resources, to use, and end-of-life. Quantification of the environmental impact of a material is not easy to assess. One approach to identify this driving force is the so-called IPAT formula, defined by Ehrlich and Holdren in 1971 [2]. The environmental impact (*I*) is the product of population (*P*), affluence (*A*) and a technology term (*T*) where *T* is understood as the reciprocal of the efficiency or material productivity (*T* = 1/*e*). Population is still increasing, and economic development is usually accompanied by an increase in material productivity.

Materials can be man-made from natural resources or recycled from waste. The performance of materials, e.g., mechanical properties or maximum service lifetime, can be improved. This can be attained, for example, by using coatings to protect against corrosion, by additives to enhance stability and processability, or by the establishment of composites.

This Special Issue, ‘Measurement of the Environmental Impact of Materials’, is focused on the impact that materials have on the environmental compartments of soil, water and air. Leaching and emission processes, including underlying mechanisms, are a recurring topic in most published articles in the present Special Issue. Contributions have come from three continents and numerous countries (USA, Germany, France, Latvia, Poland, Russia, Japan, Korea and Thailand) indicating the global dimension of the subject.

The release of substances from materials due to contact with water has been simulated and quantified by the analysis of eluates for various materials, such as polymer-based materials and primary or secondary construction materials and reported in this Special Issue. Emissions into the environment caused by leaching can affect surface waters, groundwater and soils. Some papers show that, notably, it is not sufficient to refer to the solid matter content of harmful substances, and that it is necessary to consider the processes that lead to their release. Thereby, not only chemical processes such as simple dissolution take place, but also physical mechanisms such as sorption/desorption and diffusion. The mobility of very fine particles, e.g., colloids or nanoparticles, must also be taken into account.

The environmental impact in the use phase of materials and products can be simulated using laboratory experiments that consider relevant use scenarios under accelerated conditions. A better understanding of the material’s underlying processes can be obtained when these tests are combined with exposure experiments using artificial weathering corresponding to field conditions. It is very important to study the complex and dynamic leaching processes of substances from various materials to enable regulators to set limit values and allow producers to modify their products in order to minimize the release of hazardous substances. Different exposure tests and mass transfer processes for polymer materials are reviewed in the article from Bandow et al. [3]. Weiler et al. studied the leaching behavior of irrigated building structures made of carbon-reinforced concrete with accelerated simulations at a laboratory scale [4]. Evaluation concepts are discussed in the second part of the work [5] to align simulation results to field conditions and enable the establishment of threshold values.

Construction products in contact with water and soil are subject to the possible influence of leachates on organisms. Ecotoxicological tests are becoming increasingly established as tools for understanding the impact of released hazardous substances from materials to the environment. An example of a test needing only moderate time and effort was given by von Wolff and Stephan [6], using the observation of the reproduction of worms under the influence of leachates from construction products.

Another aspect of environmental simulations regarding materials used in construction products is the need for the acquisition of reliable data on pollutant emissions, particularly regarding volatile organic compounds (VOC) to indoor air. Appropriate testing procedures are required for this purpose, in order to enable a comparable basis for the declaration of the environmental performance of products (e.g., for CE marking) by the producers. Emissions to indoor air from natural building materials using large emission chambers were studied by Richter et al. [7], and were found to be uncritical. However, the case of formaldehyde emissions from wooden toys investigated by Even et al. was different [8]. Here, the use of miniaturized test chambers was effective for the exposure assessment.

Materials may have a certain function in the environment. For example, the application of zeolitic materials as soil conditioners and as slow-release fertilizers was investigated by Szatanik-Kloc et al. [9]. It was found that low doses had a negligible effect only and the functionality has obviously been overestimated so far. Products for coastal protection can be produced from geosynthetic materials. This offers advantages such as low weight and a long service lifetime, but also presents the risk to the environment of introducing an additional source of microplastic particles. This was studied in the article by Scholz et al. [10]. The main factor influencing the ageing of geotextiles was identified, and a half-life of 330 years for the loss of 50% of the strain was calculated from their simulations. Additionally, no harmful ecotoxicological effects by leachates were observed.

A technically important function of magnesium-based alloys can be the storage of hydrogen in sustainable energy systems. The environmental burden of CaO addition to enhance performance was evaluated through a life cycle assessment (LCA) by Shin et al. [11]. More research is necessary in order to identify materials that increase performance, and the consideration of their environmental impacts should be in the foreground.

Key aspects of a sustainable circular economy have been addressed in some of the papers. The environmental impact of raw material extraction from natural deposits can be lowered when waste materials are used instead of new materials being constantly required. An example of this is the recycling of seafood waste to mono- and tricalcium phosphates [12].

One of the largest waste streams in industrialized countries originates from construction and demolition waste (CDW). The wet processing of CDW allows the utilization of the main fractions of the treatment as a substitute for natural sand and gravel [13] because pollutants are enriched in smaller sized fractions. Environmental compatibility was tested with column leaching tests, which were found to be useful for characterization as well as for quality control.

Leaching tests represent a common thread in the submissions to this Special Issue [3,4,5,6,13,14,15,16]. The underlying mass transfer principles of column leaching tests are discussed by Liu et al. [14], and Sakanakura et al. [15] showed that adsorption parameters can be determined in batch leaching tests with varying liquid–solid ratios. Ash from sewage sludge incineration (SSA) could partly replace phosphate from natural resources such as fertilizer components. However, sewage sludge contains pollutants which are transferred to the ash. Meisterjahn et al. [16] investigated the leaching of engineered nanomaterials from SSA which could be a component of sewage sludge as a result of residues from consumer products in waste water.

The direct environmental impact of materials can be evaluated by determining the release of hazardous substances during production, use, or in the end-of-life phase. Suitable measurement and testing methods for the assessment of this impact are available, as shown in many of the papers. The indirect impact of materials to the environment is caused by associated energy consumption, all emissions during manufacturing, and the depletion of natural resources. The quantification of this impact is complex but can be assessed by LCA. The sink function of nature for all negative impacts, and thus the ecosystem service [17], is limited. Hence, the use of recycled waste materials may be advantageous, in this respect, when these materials are properly characterized regarding their environmental compatibility.

It is a materials world. The rise in global material consumption will only continue in the future. Therefore, the identification of environmentally benign materials is indispensable for regulators, manufacturers, and users.
